# Supplementary material for: High-Throughput Sequencing of Complementarity Determining Region 3 in the Heavy Chain of B-Cell Receptor in Renal Transplant Recipients: A Preliminary Report
Source: J Clin Med. 2022 May 25;11(11):2980. doi: 10.3390/jcm11112980 (PMC9181060; doi:10.3390/jcm11112980)
Supplement: Supplementary file 1 [file jcm-11-02980-s001.zip › Supplementary Table S1.pdf]

**Table S1.** Clinical data in serum biochemistry, immune status, immunosuppressive medications and graft rejection, intercurrent infections or polyoma viral infection along the course of renal transplantation in 14 patients

| Patient | Time | BUN | CREA | PRA-I | PRA-II | MICA | CD3 | CD4 | CD8 | CD19 | CD16+56 | Activated T cell (%) | CD4-/CUMM | FK | CSA | Rapa | MMF/D | Predsolone | R | I | P |
|---|---|---|---|---|---|---|---|---|---|---|---|---|---|---|---|---|---|---|---|---|---|
| 1 | 0m | 73 | 11.43 | 56.3 | Neg | Neg | 86.4 | 63.7 | 20.2 | 0.1 | 12.6 | 25.5 | 490 | | | | | | | | |
| | 1m | 21 | 0.97 | 51.5 | Neg | Neg | 87.6 | 65.9 | 20 | 0 | 11.9 | 20.4 | 654 | 7.9 | | | 1000 | 5 | - | | |
| | 3m | 22 | 1.35 | 59.7 | Neg | Neg | 91.7 | 67.9 | 21.4 | 0 | 8.1 | 14.6 | 570 | 5.6 | | | 1000 | | | + | + |
| | 12m | 31 | 1.33 | 47.7 | Neg | Neg | 85.7 | 61.4 | 22.7 | 0.1 | 13.6 | 18.4 | 586 | 2.7 | | 4.5 | 500 | 5 | | | |
| 2 | 0m | 33 | 8.26 | Neg | Neg | - | - | - | - | - | - | - | - | | | | | | | | |
| | 1m | 29 | 3.58 | - | - | - | - | - | - | - | - | - | - | 5.2 | | | | | - | - | - |
| | 3m | 27 | 2.64 | - | - | - | - | - | - | - | - | - | - | 4.9 | | | | 5 | | | |
| | 12m | 29 | 2.61 | Neg | 3.9 | Neg | 79.4 | 56 | 19.1 | 8.8 | 11.1 | 12.5 | 391 | 5.4 | | | | 2.5 | | | |
| 3 | 0m | 34 | 5.98 | Neg | Neg | - | - | - | - | - | - | - | - | | | | | | | | |
| | 1m | 36 | 1.91 | - | - | - | - | - | - | - | - | - | - | 9.5 | | | 1500 | 10 | - | - | - |
| | 3m | 54 | 2.55 | - | - | - | - | - | - | - | - | - | - | 9.2 | | 1 | 1000 | 5 | | | |
| | 12m | 38 | 1.32 | Neg | Neg | Neg | 81 | 28.7 | 48.7 | 3.9 | 14.8 | 40.2 | 357 | 5.1 | | 6.1 | 1000 | 2.5 | | | |
| 4 | 0m | 73 | 6.19 | Neg | 17.8 | Neg | 93.4 | 63.7 | 29.8 | 0.2 | 5.9 | 22.6 | 680 | | | | | | | | |
| | 1m | 22 | 1.08 | Neg | Neg | Neg | 96.4 | 73.6 | 24 | 0 | 3.3 | 17.9 | 376 | 3 | | | | 5 | - | + | - |
| | 3m | 46 | 1.93 | Neg | Neg | Neg | 81.1 | 47.6 | 33.1 | 0 | 18.6 | 29.1 | 296 | 7.9 | | | 500 | 5 | | | |
| | 12m | 30 | 1.28 | Neg | Neg | Neg | 84.5 | 46.9 | 37.5 | 2.7 | 12.7 | 32.3 | 552 | 6 | | | 500 | 5 | | | |
| 5 | 0m | 30 | 6.32 | Neg | Neg | Neg | - | - | - | - | - | - | - | | | | | | | | |
| | 1m | 20 | 0.97 | - | - | - | - | - | - | - | - | - | - | 6.1 | | | 1500 | 5 | | | - |
| | 3m | 17 | 0.86 | - | - | - | - | - | - | - | - | - | - | 6.3 | | | 1500 | 5 | | + | |
| | 12m | 33 | 1.74 | Neg | Neg | Neg | 50.1 | 14 | 30.4 | 8.2 | 40.8 | 30.7 | 37 | 7.7 | | | 2000 | 10 | | | |
| 6 | 0m | 34 | 6.22 | Neg | Neg | Neg | 86.2 | 49.4 | 34.6 | 6.3 | 6.8 | 2.4 | 511 | | | | | | | | |
| | 1m | 14 | 0.84 | - | - | - | 96.9 | 65.2 | 29.4 | 0.1 | 2 | 3 | 915 | 7.4 | | | 1500 | 5 | | - | |
| | 3m | 19 | 1.29 | - | - | Neg | 98.1 | 61.7 | 32.9 | 0.2 | 1.1 | 2.6 | 1129 | 7.1 | | | 750 | 5 | | | + |
| | 12m | 52 | 7.68 | Neg | Neg | Neg | 89.7 | 46.1 | 40.5 | 0.3 | 9.1 | 5.9 | 287 | | <25.0 | | | 5 | | | |
| 7 | 0m | 95 | 16.73 | Neg | Neg | Neg | 84.1 | 58.5 | 20.8 | 5.9 | 9.7 | 4.7 | 754 | | | | | | | | |
| | 1m | 36 | 2.08 | Neg | Neg | Neg | 88.9 | 67.1 | 19.8 | 5.9 | 4.2 | 4.9 | 395 | 9.1 | | | 1500 | 15 | | | - |
| | 3m | 22 | 1.51 | Neg | Neg | Neg | 88.2 | 50.9 | 35 | 2.8 | 8.5 | 18.5 | 588 | 3 | | | 1000 | 10 | | + | |
| | 12m | 18 | 1.55 | Neg | Neg | Neg | 93.3 | 52.7 | 36.5 | 2.8 | 3.5 | 16.9 | 791 | 4.6 | | | 1000 | 10 | | | |
| 8 | 0m | 37 | 5.28 | 8.7 | Neg | Neg | 73.9 | 35.9 | 36.6 | 14.7 | 11.1 | 16.9 | 839 | | | | | | | | |
| | 1m | 16 | 1.22 | 7.6 | Neg | Neg | 74.2 | 37.4 | 36.5 | 19.5 | 6 | 10.4 | 1389 | 7.1 | | | 1500 | 10 | - | + | - |
| | 3m | 9 | 1 | 7.1 | Neg | Neg | 78.4 | 41.4 | 36.5 | 13.9 | 7.5 | 12.2 | 866 | 6.5 | | | 1500 | 2.5 | | | |
| | 12m | 8 | 0.96 | 13.1 | Neg | Neg | 79.6 | 36.4 | 41 | 11.4 | 8.4 | 26 | 850 | 7.1 | | | 1500 | 2.5 | | | |
| 9 | 0m | 57 | 10.48 | Neg | Neg | Neg | 76.4 | 43 | 29.3 | 8.6 | 14.8 | 16.6 | 533 | | | | | | | | |
| | 1m | 16 | 4.04 | Neg | Neg | Neg | 82.6 | 49.7 | 29 | 12.5 | 3.9 | 12.5 | 503 | 7.7 | | | 500 | 10 | | - | - |
| | 3m | 30 | 1.03 | Neg | Neg | Neg | 83.1 | 47.1 | 33.4 | 7.1 | 9.3 | 21.4 | 259 | 5.5 | | | 1500 | | | | |
| | 12m | - | - | - | - | - | - | - | - | - | - | - | - | | | | | | | | |
| 10 | 0m | 49 | 7.46 | Neg | Neg | Neg | 76.4 | 46.1 | 26.1 | 11.9 | 11 | 14.8 | 1002 | | | | | | | | |
| | 1m | 28 | 0.99 | Neg | Neg | Neg | 75.6 | 52 | 22.5 | 21.3 | 2 | 9.7 | 1045 | 7.1 | | | 1500 | 5 | - | - | |
| | 3m | 26 | 0.79 | Neg | Neg | Neg | 82.9 | 52 | 29.4 | 13.6 | 2.9 | 15.5 | 1656 | 6.1 | | | 1000 | 5 | | | + |
| | 12m | 18 | 0.84 | Neg | Neg | Neg | 75.9 | 46.4 | 26.8 | 18 | 5.4 | 16.2 | 1374 | 8.5 | | | 1000 | 2.5 | | | |
| 11 | 0m | 41 | 5.32 | Neg | Neg | Neg | 70.1 | 34.5 | 34.3 | 12.1 | 17 | 19.1 | 409 | | | | | | | | |
| | 1m | 23 | 1.07 | Neg | Neg | Neg | 71.2 | 37.5 | 32.3 | 22.8 | 5.6 | 10 | 730 | 6.9 | | | 1250 | 5 | - | - | + |
| | 3m | 23 | 1.08 | Neg | Neg | Neg | 75.4 | 42 | 32.3 | 17.3 | 6.9 | 13.1 | 844 | 8.2 | | | 1000 | 2.5 | | | |
| | 12m | 22 | 1.17 | Neg | Neg | Neg | 60.8 | 22.2 | 36.9 | 10.8 | 27.6 | 26 | 485 | 9.1 | | | 750 | | | | |
| 12 | 0m | 39 | 8.67 | Neg | Neg | Neg | 72.5 | 34.1 | 37.8 | 4.6 | 22.4 | 29.3 | 345 | | | | | | | | |
| | 1m | 23 | 1.67 | Neg | Neg | Neg | 81.2 | 48.7 | 33.8 | 11.8 | 6.7 | 24.4 | 327 | 4.4 | | | 1500 | 20 | | - | - |
| | 3m | 16 | 1.61 | Neg | Neg | Neg | 71.4 | 31.2 | 39.4 | 6.5 | 21.7 | 30.1 | 288 | 11.8 | | | 1500 | 15 | | | |
| | 12m | 21 | 1.92 | Neg | Neg | Neg | 75.4 | 28 | 46 | 2.9 | 21 | 32.3 | 261 | 4.7 | | | 1500 | 5 | | | |
| 13 | 0m | 43 | 7.13 | Neg | Neg | Neg | 94.5 | 41.4 | 46 | 2.2 | 2.7 | 18.1 | 703 | | | | | | | | |
| | 1m | 17 | 1.41 | Neg | Neg | Neg | 95.2 | 43.4 | 47.2 | 2.6 | 1.5 | 22 | 1052 | 7.1 | | | 1500 | 15 | - | + | - |
| | 3m | 13 | 1.01 | Neg | Neg | Neg | 95.7 | 45.3 | 45.6 | 2.3 | 1.4 | 18.2 | 1080 | 6.2 | | | 1000 | 10 | | | |
| | 12m | 14 | 0.74 | Neg | 21.7 | Neg | 92.1 | 43 | 43.3 | 4.6 | 2.4 | 14.2 | 720 | 4.8 | | | 1000 | 2.5 | | | |
| 14 | 0m | 47 | 10.39 | Neg | Neg | Neg | 76 | 37.7 | 36.7 | 12.4 | 11.4 | 20.7 | 430 | | | | | | | | |
| | 1m | 58 | 2.19 | Neg | Neg | Neg | 70.1 | 37.8 | 31.7 | 24.5 | 5 | 12.9 | 504 | 8.9 | | | 1500 | 15 | | | |
| | 3m | 24 | 1.61 | Neg | Neg | Neg | 86.6 | 49.2 | 36.3 | 8.1 | 5 | 16.8 | 522 | 7.1 | | | 1250 | 10 | | + | + |
| | 12m | 27 | 1.96 | Neg | Neg | Neg | 74.9 | 33.4 | 37.3 | 10.4 | 14.4 | 21.5 | 155 | 7.6 | | Arava 20mg | 500 | 5 | | | |

0m = D0, 1m = D1, 3m = D2, 12m = D3; BUN = blood urea nitrogen; CREA = serum creatinine; PRA = panel reactive antibody; MICA = major histocompatibility antigen class 1 chain related alloantibodies; CUMM = cubic millimeter; CD = cluster of differentiation; R = rejection; I = infection; P = polyoma viral infection; FK: tacrolimus blood concentration; Rapa: sirolimus blood concentration; MMF: mycophenolate mofetil, D = day; Prednisolone column indicates daily dose (mg); Sample number designated to the patient number is described in the Methods.
