# Supplementary material for: High-Throughput Sequencing of Complementarity Determining Region 3 in the Heavy Chain of B-Cell Receptor in Renal Transplant Recipients: A Preliminary Report
Source: J Clin Med. 2022 May 25;11(11):2980. doi: 10.3390/jcm11112980 (PMC9181060; doi:10.3390/jcm11112980)
Supplement: Supplementary file 1 [file jcm-11-02980-s001.zip › Supplementary Table S2.pdf]

Table S2. The frequency of shared profile containing 544 IGH CDR3 sequences as represented by read number of individual amino acid sequence

CDR3	ARDLDY	ARGDY	AREDY	ARGFDY	ARGDWFDP	ARDSSGWYYFDY	ARDFDY
6-0	0	0	0	0	0	0	0
1-0	0	0	0	0	0	0	0
3-0	173	0	130	173	0	147	0
4-0	0	0	0	114	0	0	0
2-0	273	0	140	0	0	118	0
5-0	114	128	335	0	135	0	0
14-0	188	320	0	706	376	0	1102
11-0	735	143	363	0	0	0	185
13-0	410	0	136	312	0	0	281
12-0	0	133	0	0	261	0	164
15-0	0	378	0	464	249	128	954
18-0	0	0	0	0	0	0	0
17-0	0	173	0	0	796	496	0
19-0	2143	0	1242	0	0	663	0

Amino acid designation; A: alanine; R: arginine; D: aspartic acid; L; leucine; Y: tyrosine; E: glutamic acid; G: glycin e Samples are designated as "sample number-time of sample taken", i.e., 1-0 = sample number 1 (from patient #1) taken at time zero

ARIGYSSSSFDY	ARGWFDP	ARGGYYFDY	AREGY	ARDRGYFDY	ARDLYYYYGMDV
0	0	0	0	0	0
6043	0	0	0	0	0
0	0	0	0	0	286
0	0	0	0	0	0
0	873	0	111	0	0
0	0	199	0	313	0
0	103	226	0	0	141
507	0	0	0	295	211
0	334	0	426	243	0
103	0	0	261	0	0
3189	197	0	171	163	0
0	0	0	0	0	0
0	0	634	0	0	0
0	0	180	0	0	512

ne F: phenylalanine; W: tryptophan; P: proline; S: serine; I: isoleucine; M: methionine; V: valine; Q: glutamine; K: l

taken at time zero.

of individual amino acid sequence.

ARDDY	ARDDAFDI	AKDSGSYYFDY	VKGGWLDY	ATSRTFDY	ASGSYYFDY	ASGFDY
0	0	0	0	0	0	0
0	0	0	390	0	0	0
0	0	0	251	0	104	130
0	0	131	0	0	166	0
0	0	133	140	0	0	0
0	178	0	0	0	0	0
1158	461	461	0	0	0	0
532	0	0	0	253	169	507
525	0	0	0	114	0	0
0	103	0	0	0	0	315
404	429	498	0	0	0	0
0	0	0	0	0	0	0
0	0	0	0	20582	0	0
0	0	0	0	0	0	0

ysine; N: asparagine; C: cysteine; H: histidine; T: threonine.

ARVGFDY	ARVDY	ARVAAAGFDY	ARSPDY	ARSDY	ARRGY	ARGYYYYGMDV
0	0	0	0	0	0	0
0	0	0	0	0	0	0
0	0	0	147	0	225	0
0	254	0	0	210	369	0
0	0	0	0	0	0	0
0	0	0	0	0	0	156
122	1262	0	0	0	0	122
0	0	143	202	363	0	0
0	0	327	0	418	0	0
255	0	200	0	0	0	236
163	369	0	421	0	0	0
0	0	0	0	0	0	0
0	0	0	0	0	0	0
0	0	0	0	0	151	0

ARGYSSSWYYFDY	ARGYDY	ARGVWFDP	ARGSSGIDY	ARGRWFDP	ARGRGY
0	0	0	0	0	0
0	0	0	0	0	0
0	294	0	173	0	0
0	210	0	0	0	0
0	0	0	0	0	0
142	0	121	0	0	0
113	0	113	0	348	0
0	0	0	0	0	0
136	0	0	342	0	593
0	0	297	0	358	212
0	0	0	163	0	180
0	0	0	0	0	0
0	14848	0	0	0	0
0	0	0	0	208	0

ARGRGWFDP	ARGRDY	ARGPGFDY	ARGNYYDSSGYLDY	ARGLFDY
0	0	0	0	0
0	0	0	0	0
0	624	104	251	0
149	0	0	0	0
0	0	0	0	0
399	142	171	349	0
0	0	0	0	0
0	0	0	0	0
0	0	616	0	175
0	0	0	370	176
0	292	0	0	0
0	0	0	0	0
0	0	0	0	0
919	0	0	0	1356

ARGGYYYGMDV	ARGGWFDP	ARGGSSWYYFDY	ARGGDY	ARGGD
0	0	0	0	0
0	0	0	0	0
0	0	0	0	0
0	0	0	0	0
0	0	0	0	0
0	0	0	0	0
188	1026	0	0	254
0	118	262	0	0
205	0	0	251	0
188	486	103	115	0
0	0	0	0	661
0	0	0	0	0
0	0	0	0	0
0	0	587	948	208

AREYYYDSSGYFDY	AREVDY	AREFDY	AREFDP	ARDYYYYGMDV	ARDYYGMDV
0	0	0	0	0	0
0	0	0	0	0	0
0	0	0	0	0	0
0	0	0	0	0	140
0	0	0	118	0	0
0	0	0	171	0	0
0	0	226	0	0	0
185	0	0	422	211	321
0	167	190	0	175	159
0	0	0	0	109	0
137	257	627	0	0	0
0	0	0	0	0	0
0	0	0	0	0	0
312	189	0	0	0	0

ARDYYFDY	ARDYYDSSGYPDY	ARDYYDSSGYFDY	ARDYWFDP	ARDYGMDV
0	0	0	0	0
0	0	0	0	0
0	0	0	346	0
0	0	0	0	0
0	0	0	0	0
0	0	156	0	0
348	0	0	0	0
0	253	0	0	126
213	0	136	966	0
394	394	0	109	133
0	292	128	0	103
0	0	0	0	0
0	0	0	0	0
0	0	0	0	0

ARDYGDYFDY	ARDWGY	ARDSSSWYYFDY	ARDRY	ARDRNWFDP
0	0	0	0	0
0	0	0	0	0
0	0	208	0	0
272	0	0	0	0
0	0	133	0	0
0	171	0	0	0
0	489	226	414	0
0	0	0	287	355
829	0	0	0	167
0	0	0	0	0
137	0	0	111	0
0	0	0	0	0
0	8791	0	0	0
0	0	0	0	2247

ARDRGYYYYYGMDV	ARDRGYSYGYFDY	ARDRGGSFDY	ARDRGDAFDI	ARDRAFDI
0	0	0	0	0
0	0	0	0	0
138	0	0	0	0
0	281	158	0	0
0	970	0	0	0
0	0	0	0	0
0	0	0	244	141
143	0	236	194	617
0	152	715	0	0
109	0	0	668	334
0	0	0	0	0
0	0	0	0	0
0	0	0	0	0
0	0	0	0	0

ARDPYYYDSSGYFDY	ARDLYGMDV	ARDLNY	ARDLGSFDY	ARDLGGYFDY
0	0	0	0	0
0	0	0	0	0
0	0	0	0	0
0	0	0	0	0
0	192	0	0	0
0	0	0	292	0
235	103	0	197	348
169	0	160	0	0
0	144	0	0	190
0	0	145	182	115
0	0	0	0	0
0	0	0	0	0
0	0	0	0	0
1356	0	123	0	0

ARDLGDY	ARDLGD	ARDIDY	ARDGGSYYFDY	ARDGGSYFDY	ARDGFDY
0	0	0	0	0	0
0	0	0	0	0	0
0	0	0	0	0	0
0	0	518	536	105	0
0	148	0	0	0	0
199	0	0	0	0	121
169	160	0	0	0	0
0	0	101	515	0	718
403	175	0	0	525	0
0	0	0	0	176	109
0	0	429	0	0	0
0	0	0	0	0	0
0	0	0	0	0	0
0	0	0	123	0	0

ARDDYGDYYFDY	ARDDYGDY	ARDDSSGYFDY	ARAVDY	ARAFDY	ARAAAAYFDY
0	0	0	0	0	0
0	0	0	0	0	0
0	0	0	0	0	0
0	0	342	0	0	131
0	0	0	0	0	0
0	0	0	0	0	0
0	0	0	0	0	0
0	143	498	473	0	0
121	114	220	334	312	0
188	0	0	0	170	631
0	326	0	713	498	111
0	0	0	0	0	0
0	0	0	0	0	0
540	0	0	0	0	0

AKVGGY	AKDSSSWYYFDY	AKDRYSSGWYYFDY	AKDLDY	AKDGDY	AKDFDY
0	0	0	0	0	0
0	0	0	0	0	0
0	0	130	130	0	0
0	483	140	158	114	0
133	0	0	0	0	0
0	0	0	0	0	0
0	0	0	0	0	0
0	0	0	0	0	0
0	0	0	235	152	471
133	328	0	0	291	145
0	103	309	0	0	567
0	0	0	0	0	0
0	0	0	0	0	0
246	0	0	0	0	0

AKDDY	VRRGDGYKYDY	VRDMDV	VRDHNWGFDY	TTRSDY	TTGFGNGEAH
0	0	0	0	0	0
0	0	0	0	0	0
0	0	0	606	0	0
0	0	0	0	0	0
0	0	385	0	0	0
0	0	0	0	0	0
320	0	103	0	103	0
0	0	0	0	0	802
243	121	0	0	0	0
0	6306	0	1051	0	723
0	0	0	0	189	0
0	0	0	0	0	0
299	0	0	0	0	0
0	0	0	0	0	0

TRDDYYFDY	TESLIH	ATYDY	ATSSFDY	ATGYYFDY	ATGYSSSWYYFDY	ATFDY
0	0	0	0	0	0	0
0	0	0	0	0	0	0
0	0	0	0	0	0	0
0	0	0	430	0	0	0
0	0	0	0	0	0	0
0	0	0	0	0	0	0
0	339	0	0	0	235	0
0	287	0	0	0	0	0
0	0	479	616	205	0	357
188	0	273	0	0	0	0
0	0	0	0	0	206	2501
0	0	0	0	0	0	0
0	0	0	0	0	0	0
407	0	0	0	3945	0	0

ATDAGGGDY	ASSSGWYFDY	ASRDY	ASPKGP	ASPFDY	ASLNWFDP	ASLGDY
0	0	0	0	0	0	0
0	0	0	0	0	0	0
121	0	0	0	0	0	0
0	0	0	0	0	369	0
1073	103	0	0	0	0	0
0	0	0	0	0	0	0
0	0	471	913	0	0	527
0	0	0	363	0	0	0
0	0	723	0	0	0	0
0	0	0	0	157	230	279
0	507	0	0	369	0	0
0	0	0	0	0	0	0
0	0	0	0	0	0	0
0	0	0	0	0	0	0

ASHGY	ASGSYFDY	ASGSGYQYYFDY	ASEDY	ARYTYGFDY	ARYSYGFDY
0	0	0	0	0	0
0	0	0	0	0	0
294	0	0	0	0	0
0	0	0	0	0	0
0	0	0	0	0	0
0	0	0	0	0	0
0	0	339	386	0	0
0	422	0	0	160	0
243	0	0	0	0	0
0	1160	0	0	261	182
0	0	0	0	0	103
0	0	0	0	0	0
0	0	0	0	0	0
0	0	132	208	0	0

ARYSSSWYYFDY	ARYSSGWYYFDY	ARWNEGLDY	ARWDY	ARVYY
0	0	0	0	0
0	0	0	0	0
0	0	0	0	112
0	0	0	0	0
0	0	0	2895	0
0	0	0	0	0
0	113	160	0	0
152	0	0	0	0
0	0	0	0	0
151	1998	0	164	115
0	0	0	0	0
0	0	0	0	0
0	0	0	0	0
0	0	8677	0	0

ARVYDSSGYYYYYYGMDV	ARVVPAAIFDY	ARVVGPFDY	ARVTYYYDSSGYYWFDP
0	0	0	0
0	0	390	0
0	0	0	0
0	0	0	0
0	0	0	0
0	0	427	0
0	0	0	0
169	109	0	109
0	167	0	0
0	0	0	0
0	0	0	0
0	0	0	0
0	0	0	2307
844	0	0	0

ARVSVAGDDY	ARVSSSWYYFDY	ARVSNWFDP	ARVRTYYYGMDV
0	0	0	0
0	0	0	0
0	0	0	0
131	158	0	0
0	0	0	0
0	0	0	1183
103	0	0	0
0	0	0	0
0	129	0	144
0	0	1853	0
0	0	0	0
0	0	0	0
0	0	1765	0
0	0	0	0

ARVRSGSYYFDY	ARVRDAFDI	ARVLDDAFDI	ARVLAAAGTGWFDP	ARVKSWFDP
0	0	0	0	0
0	0	0	0	0
147	0	112	0	0
0	105	0	0	0
0	0	0	0	0
0	0	0	0	249
0	0	0	0	113
0	160	0	152	0
0	0	0	0	0
0	0	0	0	0
0	0	137	266	0
0	0	0	0	0
0	0	0	0	0
900	0	0	0	0

ARVHYYYYGMDV	ARVGYYYGMDV	ARVGYTHWSIDD	ARVGYGDYDFDY
0	0	0	0
0	0	0	0
0	0	0	0
0	0	0	105
0	0	0	0
0	0	0	0
244	0	169	0
0	101	0	0
114	0	0	0
0	0	0	164
0	464	1005	0
0	0	0	0
0	0	0	0
0	0	0	0

ARVGGGSYFDY	ARVGGAFDI	ARVGATTDAFDI	ARVAAAGYYYYGMDV
0	0	0	0
0	0	0	0
0	0	0	0
0	0	0	0
0	0	0	0
0	0	0	0
226	0	0	122
0	0	101	0
205	144	555	0
0	0	0	0
0	0	0	189
0	0	0	0
0	0	0	0
0	1156	0	0

ARTYYDFWSGYFDY	ARTNWFDP	ARTNAFDI	ARTEDYYYGMDV	ARTDY
0	0	0	0	0
0	0	0	0	0
0	0	0	0	0
0	0	0	0	0
0	0	0	0	0
0	0	0	0	0
169	0	0	0	0
236	109	202	0	219
0	0	0	152	0
0	0	407	0	0
0	180	0	0	154
0	0	0	0	0
0	0	0	149	0
0	0	0	0	0

ARSYYDSSGYYYFDY	ARSYDILTGYYYY	ARSVGYSSGWYDY	ARSSYYYYGMDV
0	0	0	0
0	0	0	0
286	0	0	0
0	0	219	0
0	0	0	0
0	0	0	0
0	0	0	0
0	0	0	380
0	0	0	0
413	182	151	0
0	0	0	0
0	0	0	0
0	0	0	0
0	360	0	170

ARSSGYYYGY	ARSRRDGYNWDY	ARSPFDY	ARSIVGATTLDY	ARSIAAAGNWFDP
0	0	0	0	0
0	0	0	0	0
0	0	0	0	0
0	149	0	0	0
0	0	0	0	0
0	0	0	328	0
0	0	0	0	0
177	0	0	0	0
0	190	0	0	0
388	0	157	170	145
0	0	0	0	232
0	0	0	0	0
0	0	2007	0	0
0	0	0	0	0

ARSAGAFDI	ARSAFDI	ARRYSSGWYYFDY	ARRYFDL	ARRYCSSTSCLFDY	ARRTY
0	0	0	0	0	0
0	0	0	0	0	0
0	0	0	0	0	0
0	606	0	131	0	0
125	0	0	0	0	0
0	0	0	0	0	0
0	0	197	0	169	0
0	160	169	118	0	321
0	0	0	0	0	0
0	0	0	0	0	0
447	0	0	0	0	644
0	0	0	0	0	0
0	0	0	0	484	0
0	0	0	0	0	0

ARRRCSSTSCFFDY	ARRNYYYYGMDV	ARRGSGGSFDY	ARPYYYYGMDV
0	0	0	0
0	0	0	0
0	0	0	0
0	0	0	0
0	0	0	0
0	0	185	0
0	0	0	0
0	0	143	135
547	266	0	0
249	0	0	121
0	240	0	0
0	0	0	0
0	0	0	0
0	0	0	0

ARPYCSGGSCYFDY	ARPFDY	ARNYYDSSGYYPFDY	ARNDAFDI	ARLSSSWYYFDY
0	0	0	0	0
0	0	0	0	0
0	0	0	0	0
0	377	0	0	0
0	0	0	0	0
0	0	0	0	271
141	0	0	0	348
0	0	0	0	0
0	0	0	0	0
127	0	133	212	0
0	0	0	412	0
0	0	0	0	0
0	0	0	0	0
0	1384	663	0	0

ARLRGGYDFDY	ARLRAAAGYYFDY	ARLNWFDP	ARKFDP	ARIGGSYGY	ARIDY
0	0	0	0	0	0
0	0	0	0	0	0
0	0	0	0	0	0
0	272	650	0	0	0
162	0	0	0	0	140
0	0	0	0	0	0
0	282	0	0	0	0
185	0	0	0	0	0
0	0	0	410	1423	0
0	0	236	455	315	133
0	0	0	0	0	0
0	0	0	0	0	0
0	0	0	0	0	0
0	0	0	0	0	0

ARHYDY	ARHRVPDY	ARHGGSSWYFDY	ARGYYYYYMDV	ARGYYFDY
0	0	0	0	0
0	0	0	0	0
0	0	0	0	0
0	0	0	0	0
0	0	11803	0	0
0	213	0	0	0
0	282	0	216	113
0	0	0	228	0
0	0	0	0	570
388	0	0	0	0
137	0	472	0	0
0	0	0	0	0
0	0	0	0	0
0	0	0	0	0

ARGYYDSSGYLFDY	ARGYWFDP	ARGYSYGYFDY	ARGYSSGWYYFDY
0	0	0	0
0	0	0	0
0	182	0	0
114	0	166	0
0	0	0	0
0	0	0	0
0	0	508	0
0	0	0	481
0	0	0	251
164	0	0	0
0	103	0	0
0	0	0	0
0	0	0	0
0	0	0	0

ARGYSSGWYDY	ARGYSGYFDY	ARGYGMDV	ARGYFDY	ARGYDSSGYYFDY
0	0	0	0	0
0	0	0	0	0
0	0	0	0	0
0	131	0	0	0
0	0	0	0	0
228	0	0	0	135
0	0	1149	216	0
0	0	219	0	0
228	0	0	0	0
0	0	0	0	1603
0	103	0	352	0
0	0	0	0	0
0	0	0	0	0
0	0	0	0	0

ARGYCSSTSCYYFDY	ARGYCSSTSCLDY	ARGYCSGGSCYPFDY	ARGYCSGGSCYPDY
0	0	0	0
0	0	0	0
0	121	0	121
0	0	0	369
0	0	0	0
0	0	142	0
0	0	0	0
295	177	0	0
0	0	0	0
176	0	0	0
0	0	120	0
0	0	0	0
0	0	0	0
0	0	0	0

ARGYCSGGSCYFDY	ARGYAFDI	ARGWYFDL	ARGVYYFDY	ARGVYFDY
0	0	0	0	0
0	0	0	0	0
0	0	0	0	0
0	0	0	158	140
0	0	0	0	0
0	0	0	0	0
0	0	0	0	0
253	152	0	0	397
0	0	175	547	0
0	0	0	0	0
361	128	0	0	0
0	0	0	0	0
0	0	11929	0	0
0	0	0	0	0

ARGVDYFDY	ARGVDY	ARGVAVAGFDY	ARGTYYDFWSGYPYFDY	ARGTTVTTFDY
0	0	0	0	0
0	0	0	0	0
0	0	0	0	0
0	0	0	219	0
0	0	0	0	0
0	9891	0	0	0
0	188	0	320	0
0	0	194	0	177
129	0	0	0	258
121	0	0	0	0
0	0	481	0	0
0	0	0	0	0
0	0	0	0	0
0	0	0	0	0

ARGTTDFDY	ARGTFDY	ARGTDAFDI	ARGSWFDP	ARGSVAGDY	ARGSSSWYYFDY
0	0	0	0	0	0
0	0	0	0	0	0
0	0	138	0	0	0
0	0	0	0	0	0
170	0	0	0	0	0
0	0	0	0	0	356
565	329	0	1092	0	0
0	0	473	0	169	0
0	0	0	0	159	0
0	0	0	0	0	0
0	111	0	0	0	111
0	0	0	0	0	0
0	0	0	0	0	0
0	0	0	654	0	0

ARGSSSCPFDY	ARGSSGWYYFDY	ARGSSFDY	ARGSNYGMDV	ARGSIAARRYYFDY
0	0	0	0	0
0	0	0	0	0
0	0	0	0	0
483	0	0	246	0
0	0	0	111	0
0	0	0	0	121
0	0	0	0	216
0	236	0	0	0
0	0	0	0	0
157	0	182	0	0
0	0	0	0	0
0	0	0	0	0
0	0	0	0	0
0	142	161	0	0

ARGSGSLDY	ARGSGNFDY	ARGSDY	ARGRYSYGYYFDY	ARGRSYYGY
0	0	0	0	0
0	0	0	0	0
0	0	0	0	0
0	0	166	0	0
0	0	0	0	0
0	0	0	0	164
207	226	0	584	254
0	0	0	0	0
753	0	159	0	0
0	0	0	328	0
0	283	0	0	0
0	0	0	0	0
0	0	0	0	0
0	0	0	0	0

ARGRSSGWYYYYGMDV	ARGRLYYGMDV	ARGRLYFDY	ARGRLQFDY
0	0	0	0
0	0	0	0
0	121	0	0
0	193	0	667
0	0	0	0
0	0	14463	0
0	0	0	0
0	0	0	0
121	0	129	0
0	0	0	0
326	0	0	0
0	0	0	0
0	0	0	0
0	0	0	644

ARGRLNFDY	ARGRGYSYGIDY	ARGRGYGDY	ARGRGSYPNWFDP	ARGRGDY
0	0	0	0	0
0	0	0	0	0
0	0	0	0	0
0	0	0	0	0
0	0	0	0	0
0	0	0	142	0
0	0	395	781	0
202	0	109	0	0
0	350	0	0	0
0	388	0	0	1731
0	0	0	0	0
0	0	0	0	0
0	0	0	0	0
749	0	0	0	531

ARGRDYFDY	ARGRAFDP	ARGPYCSGGSCYRLFDY	ARGPSLDY	ARGPFDAFDI
0	0	0	0	0
0	0	0	0	0
0	0	0	0	0
0	0	0	193	0
0	0	0	0	111
0	0	0	0	0
461	226	0	0	584
0	0	0	0	0
0	0	327	0	0
0	0	0	103	0
0	154	232	0	0
723	0	0	0	0
0	0	0	0	0
0	0	0	0	0

ARGPDY	ARGPDAFDI	ARGNYYYYYGMDV	ARGNYGDYFDY	ARGNWFDP
0	0	0	0	0
0	0	0	0	0
0	0	104	0	0
0	0	0	0	0
155	0	0	0	0
292	114	0	0	0
0	0	0	103	0
0	0	0	0	270
0	0	0	433	0
0	218	297	0	0
0	0	0	0	146
0	0	0	0	0
0	0	0	0	0
0	0	0	0	0

ARGNTAMDNFDY	ARGNSSGWYYYYYGMDV	ARGNPFDI	ARGNAFDI
0	0	0	0
0	0	0	0
130	0	0	0
0	0	0	0
0	0	0	0
0	0	0	149
0	0	0	0
0	101	177	0
121	350	0	0
0	0	0	0
0	0	214	103
0	0	0	0
0	0	0	0
0	0	0	0

ARGLYGMDV	ARGLSSSWYYFDY	ARGLGY	ARGLGWFDP	ARGLAAAGTIDY
0	0	0	0	0
0	0	0	0	0
0	0	0	0	0
289	0	0	0	0
0	0	0	0	0
0	0	0	0	0
0	197	0	0	0
312	0	101	0	118
0	0	471	0	654
0	0	0	0	0
0	343	0	438	0
0	0	0	0	0
0	0	0	0	0
0	0	0	597	0

ARGILSDY	ARGIDY	ARGIDFDY	ARGIAAAGYYFDY	ARGIAAAGGDY	ARGIAAAGDY
0	0	0	0	0	0
0	0	0	0	0	0
0	121	0	0	0	0
0	0	0	0	0	0
125	0	0	0	125	0
0	0	0	0	178	185
0	131	226	0	0	0
0	0	177	219	0	202
0	0	0	0	0	0
0	0	0	249	0	0
240	0	0	0	0	0
0	0	0	0	0	0
0	0	0	0	0	0
0	0	0	0	0	0

ARGHYGLDV	ARGHWFDP	ARGHFDY	ARGGYYYYGMDV
0	0	0	0
0	0	0	0
0	0	0	0
0	0	588	0
0	0	0	0
634	0	0	121
0	0	0	0
1969	0	0	152
0	251	0	0
0	0	188	0
0	128	0	0
0	0	0	0
0	0	0	0
0	0	0	0

ARGGYSYGYYYYYGMDV	ARGGYSYGYYFDY	ARGGYSSSWYYFDY	ARGGYSHGFDV
0	0	0	0
0	0	0	0
0	0	0	1092
0	0	0	0
0	111	0	0
0	0	0	0
0	0	216	0
0	380	0	0
121	0	0	0
0	0	0	0
0	0	111	593
0	0	0	0
0	0	0	0
256	0	0	0

ARGGYSGYDFDY	ARGGVYYYGMDV	ARGGVTDAFDI	ARGGSYYFDY	ARGGSYLDY
0	0	0	0	0
0	0	0	0	0
0	0	0	0	0
0	0	0	0	219
0	0	0	0	0
0	0	0	0	0
0	0	254	0	226
0	0	0	0	0
190	273	0	0	0
115	200	0	249	0
0	0	128	584	0
0	0	0	0	0
0	0	0	0	0
0	0	0	0	0

ARGGSRYFDY	ARGGNWFDP	ARGGIAAAGTFDY	ARGGGSLDY	ARGGGMDV
0	0	0	0	0
0	0	0	0	0
0	0	0	0	173
0	0	0	0	0
0	0	0	0	0
0	0	0	235	0
254	0	0	0	0
0	135	0	0	0
0	136	121	0	152
729	0	0	0	0
0	0	120	103	0
0	0	0	0	0
0	0	0	0	0
0	0	0	0	0

ARGGGDY	ARGGGAFDI	ARGGFDY	ARGGDYYYYGMDV	ARGGDYFDY
0	0	0	0	0
0	0	0	0	0
0	0	0	0	0
254	0	0	3585	0
0	0	0	0	0
0	0	0	0	0
697	131	0	0	706
0	0	101	295	0
0	0	395	0	220
0	0	0	0	0
0	584	0	0	0
0	0	0	0	0
0	0	0	0	0
0	0	0	0	0

ARGGDPGYFDY	ARGGDCSSTSCYLPFDY	ARGEYDY	ARGEGY	ARGDYYYGMDV
0	0	0	0	0
0	0	0	0	0
130	277	0	0	138
0	0	2021	0	0
0	0	0	103	0
0	0	0	0	0
0	0	0	0	0
0	118	126	0	0
266	0	0	167	464
0	0	0	0	0
0	0	0	0	0
0	0	0	0	0
0	0	0	0	0
0	0	0	0	0

ARGDYGMDV	ARGDYGFDY	ARGDYFDY	ARGDSSSWYYFDY
0	0	0	0
0	0	0	0
0	0	0	0
0	0	0	0
0	0	0	133
0	0	0	0
0	0	0	0
0	0	0	0
334	418	449	0
0	0	0	0
0	128	146	369
0	0	0	0
0	0	0	0
180	0	0	0

ARGDSSGWYYYYGMDV	ARGDPDY	ARGDGDYYFDY	ARGCGGDCYSDFDY
0	0	0	0
0	0	0	0
0	0	0	0
0	105	0	0
0	0	0	0
0	0	0	0
0	0	395	0
0	0	0	185
121	0	0	0
103	0	0	0
0	137	0	343
0	0	0	0
0	0	0	0
0	0	749	0

ARGAYYYYGMDV	ARGAYSSSWYYFDY	ARGAVAGTFDY	ARGATLIDY	ARGAPLDY
0	0	0	0	0
0	0	0	0	0
0	0	0	0	0
0	0	140	0	0
0	0	0	0	0
0	0	0	0	313
244	0	0	0	0
0	304	0	160	0
0	0	0	0	0
303	382	0	0	0
0	0	0	0	214
0	0	0	0	0
0	0	0	0	0
0	0	113	132	0

ARGAPFDY	ARGAGRFDP	ARGAFDI	ARFPARRAD	ARFHYYGMDV
0	0	0	0	0
0	0	0	0	0
0	303	658	0	0
0	0	0	0	325
0	0	0	0	0
0	0	0	242	256
442	838	0	0	0
405	0	228	0	0
0	0	0	0	0
0	0	0	0	0
0	0	0	0	0
0	0	0	0	0
0	0	0	138	0
0	0	0	0	0

AREYYDSSGYYDY	AREYSSSWYWYFDL	AREWSAFDI	AREWELTFDY	AREWELLPDY
0	0	0	0	0
0	0	0	0	0
0	0	0	0	0
0	5544	500	0	0
0	0	0	0	0
185	0	0	0	171
0	0	0	546	0
0	152	169	0	118
258	0	0	0	0
0	0	0	139	0
0	0	0	0	0
0	0	0	0	0
0	0	0	0	0
0	0	0	0	0

AREVPFDY	AREVN	AREVHGMDV	AREVGDAFDI	ARESSGLDY	ARESIAVAGTGFDY
0	0	0	0	0	0
0	0	0	0	0	0
0	0	0	0	0	0
0	0	0	0	0	0
0	0	0	0	118	0
0	0	0	0	0	0
0	0	0	235	461	0
0	0	0	0	0	0
220	0	205	0	0	159
352	0	194	0	0	0
0	197	0	128	0	111
0	0	0	0	0	0
0	0	0	0	0	0
0	265	0	0	0	0

ARESH	ARESGWYFDY	ARERRSFDY	ARERRGYYAEY	ARERGGYYFDY
0	0	0	0	0
0	0	0	0	0
0	0	0	0	0
0	0	0	0	0
0	0	0	0	0
0	0	114	0	0
0	122	141	0	207
228	101	0	0	414
0	0	0	0	0
164	0	0	1840	0
0	0	0	0	0
0	0	0	5116	0
0	0	0	0	0
0	0	0	0	0

AREREGDFDY	AREPFDY	AREPDY	ARENY	ARENHDY	ARELFDY	ARELDY
0	0	0	0	0	0	0
0	0	0	0	0	0	0
0	0	156	0	0	0	0
0	0	0	0	0	0	0
0	0	0	0	0	170	0
0	0	0	0	213	0	0
395	0	0	0	0	0	1205
245	0	0	118	0	0	0
0	182	0	0	266	0	0
0	0	0	0	0	315	267
0	128	266	0	0	0	0
0	0	0	0	0	0	0
0	0	0	0	0	0	0
0	0	0	303	0	0	0

AREGYYYGMDV	AREGYYYDSSGYYYFDY	AREGYYGSGSYSFDY	AREGYSYGSFDY
0	0	0	0
0	0	0	0
0	0	0	0
0	105	123	0
0	0	0	0
0	0	0	0
0	0	0	0
169	0	0	253
0	0	0	0
0	0	0	151
369	0	0	0
0	0	0	0
0	0	0	0
0	502	123	0

AREGYGDPDY	AREGSYFDY	AREGSSGWYFDY	AREGLNWFDP	AREGIVFDY
0	0	0	0	0
0	0	0	0	0
0	0	0	0	0
0	105	0	0	0
0	0	0	0	0
0	0	0	0	0
169	0	160	188	0
152	507	0	0	202
0	0	0	152	0
0	0	0	0	0
0	0	120	0	0
0	0	0	0	0
0	0	0	0	0
0	0	0	0	1251

AREGIAVAGFDY	AREGIAAAGTGYYYYGMDV	AREGGSYYYGMDV	AREGGSYYFDY
0	0	0	0
0	0	0	0
0	0	0	147
0	140	0	0
0	0	0	0
0	0	249	0
141	160	0	0
0	0	0	0
0	0	0	312
0	0	0	0
0	0	0	0
0	0	0	0
0	0	657	0
1507	0	0	0

AREGDFDY	AREDYYDSSGYYDY	AREDWFDP	AREDSSGWYYFDY	AREDLDY
0	0	0	0	0
0	0	0	0	0
0	147	0	0	0
0	0	0	0	0
1088	0	125	111	0
0	0	0	0	0
0	0	0	0	0
0	245	245	0	109
0	0	0	0	0
103	0	0	0	0
0	0	0	0	120
0	0	0	0	0
0	0	0	0	0
0	0	0	1137	0

AREDIVVVPAAMDY	AREDDSSGYYYY	AREDDAFDI	AREAPYYFDY	AREAHAFDI
0	0	0	0	0
0	0	0	0	0
0	0	138	0	0
0	0	0	0	0
0	0	0	0	0
0	0	0	0	0
0	141	0	235	0
177	0	0	0	228
0	258	0	388	281
188	0	0	0	0
0	0	0	0	0
0	0	0	0	0
0	0	0	0	0
0	0	559	0	0

AREAGYYFDY	AREAAGFDY	ARDYYYGMDV	ARDYYGSGSYFDY	ARDYYGSGSPDY
0	0	0	0	0
0	0	0	0	0
0	156	0	0	0
184	0	0	0	0
0	540	0	0	0
0	0	0	0	0
0	0	0	0	0
312	0	0	0	0
0	0	205	0	129
0	0	0	291	0
0	0	0	283	0
0	0	0	0	0
0	0	0	0	0
0	0	246	0	379

ARDYYDSSGYYYYYYGMDV	ARDYYDSSGYYSFDY	ARDYYDSSGYVDY
0	0	0
0	0	0
104	0	0
0	0	0
0	0	0
0	0	0
0	0	113
0	312	143
0	289	0
0	0	0
163	0	0
0	0	0
0	0	0
0	0	0

ARDYGGNSGWFDP	ARDYGDYYYYGMDV	ARDYGDY	ARDYFDY	ARDYDY	ARDVY
0	0	0	0	0	0
0	0	0	0	0	0
130	0	0	0	0	0
0	0	0	0	0	0
0	0	0	0	0	0
0	0	0	0	0	121
0	113	0	0	687	1601
0	0	0	101	0	0
0	121	182	0	464	0
0	0	0	0	0	0
0	0	0	0	0	0
0	0	0	0	0	0
2491	0	0	0	0	0
0	0	199	1346	0	0

ARDSYYYDSSGYYYFDY	ARDSYGMDV	ARDSSYYYDSSGYFDY	ARDSSSWYDAFDI
0	0	0	0
0	0	0	0
112	0	0	0
0	0	0	0
0	0	0	0
0	0	0	0
0	0	0	0
177	109	0	194
0	449	0	0
0	0	236	309
0	0	0	0
0	0	0	0
0	0	0	0
0	0	834	0

ARDSSSFDY	ARDSSGWYFDY	ARDSSGWFDY	ARDSSGFDAFDI	ARDSRDAFDI
0	0	0	0	0
0	0	0	0	0
138	0	0	138	0
0	0	0	0	0
0	0	0	0	0
0	0	0	0	0
150	0	0	0	207
0	0	295	160	0
0	220	449	0	0
0	0	0	0	0
0	137	0	0	318
0	0	0	0	0
0	0	0	0	0
0	0	0	0	0

ARDSPHFDY	ARDSIAAAGTFDY	ARDSGYYDSSGYFDY	ARDSGSYYGY	ARDSGSYFDY
0	0	0	0	0
0	0	0	0	0
0	0	0	0	0
0	0	0	0	0
0	0	0	0	0
0	0	0	0	0
179	0	0	320	310
0	0	0	0	0
662	0	0	0	601
0	145	218	0	0
0	0	0	137	0
0	0	0	0	0
0	0	0	0	0
0	796	113	0	0

ARDSGSYDY	ARDSGAFDI	ARDSDY	ARDSAFDI	ARDRYYYDSSGYYYYYYGMDV
0	0	0	0	0
0	0	0	0	0
0	0	0	0	147
0	0	0	0	0
0	0	0	0	0
0	0	292	0	0
0	650	0	0	0
0	0	693	219	0
342	0	0	0	0
0	0	0	0	218
0	0	0	120	0
0	0	0	0	0
0	0	0	0	0
749	1422	0	0	0

ARDRYYYDSSGYFDY	ARDRYSSSWYYYYGMDV	ARDRYGMDV	ARDRYGAFDI
0	0	0	0
0	0	0	0
104	0	0	0
0	0	0	0
0	0	0	0
0	0	0	0
0	188	169	0
0	0	0	363
0	213	0	190
0	0	0	0
0	0	0	0
0	0	0	0
0	0	0	0
597	0	256	0

ARDRYFDY	ARDRVGGMDV	ARDRSWFDP	ARDRSSSSLDY	ARDRSLDY	ARDRSAFDI
0	0	0	0	0	0
0	0	0	0	0	0
0	112	173	0	0	0
114	0	0	0	219	0
0	0	0	0	0	0
0	0	0	0	0	0
0	0	0	320	358	188
0	380	0	312	0	0
0	0	190	0	0	0
0	0	0	0	0	0
163	0	0	0	0	223
0	0	0	0	0	0
0	0	0	0	0	0
0	0	0	0	0	0

ARDRRYYYDSSGYSSFDY	ARDRRYDYFDY	ARDRNYYFDY	ARDRLFDY
0	0	0	0
0	0	0	0
0	0	0	0
0	0	0	0
0	0	0	177
0	0	171	0
0	0	0	0
211	160	160	118
0	570	0	0
0	0	0	0
0	0	0	0
0	0	0	0
0	0	0	0
1261	0	0	0

ARDRGWFDP	ARDRGSGWYYFDY	ARDRGDFDY	ARDRGAVPGDYFDY	ARDRGAFDI
0	0	0	0	0
0	0	0	0	0
0	112	0	0	0
0	0	0	0	0
0	0	0	1303	0
0	0	149	0	0
320	0	0	179	0
693	0	0	0	0
0	266	426	0	159
0	0	0	0	212
0	0	0	0	0
0	0	0	0	0
0	0	0	0	0
0	0	0	0	0

ARDRGAAADYYYYGMDV	ARDRDDYFDY	ARDRDAFDI	ARDRCSGGSCYSDY
0	0	0	0
0	0	0	0
0	0	0	0
0	0	0	0
0	0	0	0
0	0	0	0
131	0	235	0
0	583	0	185
175	0	0	0
0	0	230	115
0	292	0	0
0	0	0	0
0	0	0	0
0	0	0	0

ARDRCSGGSCYPYYYYYGMDV	ARDRATVTFDY	ARDQYYYDSSGYSYFDY
0	0	0
0	0	0
130	199	0
0	0	0
0	0	0
0	0	0
0	0	0
135	0	118
0	0	0
0	243	0
0	0	103
0	0	0
0	0	0
0	0	0

ARDQGDY	ARDQDGLGY	ARDQDDILTGYYDY	ARDPYYYDSSGYYFDY
0	0	0	0
0	0	0	0
0	0	0	0
123	0	0	0
0	0	0	0
0	0	0	0
310	0	0	0
0	0	0	0
0	190	0	0
0	0	109	407
0	223	0	0
0	0	0	0
0	0	0	2526
0	0	1052	0

ARDPVAGLFDY	ARDPGWGAIDI	ARDPFRSSFDS	ARDPFDYGGYFDY	ARDPFDY
0	0	0	0	0
0	0	0	0	0
0	0	0	0	0
0	0	536	0	166
0	0	177	0	0
0	542	0	0	0
103	376	0	0	0
0	0	0	0	0
509	0	0	0	197
0	0	0	127	0
0	0	0	455	0
0	0	0	0	0
0	0	0	0	0
0	0	0	0	0

ARDPDY	ARDPDV	ARDPDAFDI	ARDPCGGDCYSGDY	ARDPAGDYGY
0	0	0	0	0
0	0	0	0	0
0	0	0	0	0
0	0	0	325	123
1791	162	0	0	0
0	0	0	0	0
0	0	0	0	0
0	219	304	0	0
1027	0	0	0	0
0	0	0	0	0
0	0	0	249	180
0	0	0	0	0
0	0	0	0	0
0	0	1346	0	0

ARDPAFSAFDV	ARDPAFDY	ARDPAAGTLDY	ARDNYYGMDV	ARDMDV
0	0	0	0	0
0	0	0	0	0
355	0	0	0	0
0	166	0	0	0
0	0	0	0	0
342	0	0	106	0
0	131	489	0	0
0	0	0	202	0
0	0	0	0	304
0	0	0	0	157
0	0	206	0	0
0	0	0	0	0
0	0	0	0	0
0	0	0	0	0

ARDMDAFDI	ARDLYYYDSSGYLDY	ARDLYY	ARDLYGSGSYLDY
0	0	0	0
0	0	5502	0
0	0	0	0
316	0	0	0
0	0	0	103
0	0	0	0
0	0	0	235
0	0	0	0
182	0	0	0
0	285	121	0
0	180	0	0
0	0	0	0
0	0	0	0
0	0	0	0

ARDLVGATTDWFDP	ARDLSSWYYFDY	ARDLSGSYLDY	ARDLSGPDY
0	0	0	0
0	0	0	0
0	0	0	0
0	0	0	0
0	103	0	0
0	0	0	4136
0	0	0	0
0	0	0	0
0	0	235	0
103	182	0	0
171	0	154	0
0	0	0	0
0	0	0	0
0	0	0	189

ARDLSGGGMDV	ARDLSFDY	ARDLRAFDI	ARDLNYYGMDV	ARDLNGMDV
0	0	0	0	0
0	0	0	0	0
4359	381	0	104	0
0	0	0	0	0
0	0	0	0	0
125584	0	0	0	11453
0	0	0	0	0
0	0	185	0	0
0	0	0	0	129
0	0	0	0	0
0	361	0	0	0
0	0	0	0	0
0	0	149	0	0
0	0	0	227	0

ARDLLDY	ARDLKGYYYGMDV	ARDLGYCSSTSCYSDAFDI	ARDLGPDY
0	0	0	0
0	0	0	0
0	0	0	0
0	984	0	228
0	0	0	0
0	128	0	0
376	0	0	0
0	0	0	236
0	0	152	0
0	0	0	0
5372	0	240	0
0	0	0	0
0	0	0	0
0	0	0	0

ARDLDSSSWYDY	ARDKYYFDY	ARDISWFDP	ARDILTGYYSDY	ARDIAAAGTFDY
0	0	0	0	0
0	0	0	0	0
0	0	0	0	156
0	0	0	0	0
0	0	0	0	0
0	0	0	0	0
226	169	0	0	216
0	0	0	194	0
159	0	479	0	0
0	0	0	255	0
0	0	0	0	0
0	0	0	0	0
0	0	0	0	0
0	265	550	0	0

ARDHPVAGLYFDY	ARDHGYGMDV	ARDHGDY	ARDHFGMDV
0	0	0	0
5321	0	0	0
0	121	0	0
0	0	0	166
118	0	0	0
0	0	0	0
0	0	0	0
0	0	287	0
0	0	0	0
0	0	0	267
0	0	103	0
0	0	0	0
0	0	0	0
0	1213	0	0

ARDHCSSTSCYYYYGMDV	ARDHCSGGSCYFDY	ARDGYYYYGMDV
0	0	0
0	0	0
0	0	0
0	0	0
0	0	0
0	0	0
160	0	216
0	135	0
205	0	190
0	0	0
0	0	0
0	0	0
0	0	0
0	730	0

ARDGYYYGMDV	ARDGYSSGWYYFDY	ARDGTNYYGMDV	ARDGSGSYLDY
0	0	0	0
3607	0	0	0
563	0	0	0
0	0	0	0
0	0	0	0
0	0	0	0
0	0	537	329
0	152	0	0
0	0	0	350
0	0	267	0
0	137	0	0
0	0	0	0
0	0	0	0
0	0	0	0

ARDGSAWSRDY	ARDGIAAAGSNWFDP	ARDGH	ARDGGY	ARDGGVDAFDI
0	0	0	0	0
0	0	0	0	0
0	0	0	0	0
0	351	0	0	0
1392	0	0	0	0
0	0	0	114	0
0	0	876	0	150
3431	0	0	0	101
0	0	0	365	0
0	394	176	0	0
0	0	0	0	0
0	0	0	0	0
0	0	0	0	0
0	0	0	0	0

ARDGGSYRFDY	ARDGGSNWFDP	ARDGGGYFDY	ARDGGGSYYFDY	ARDGGFDY
0	0	0	0	0
0	0	0	0	0
0	164	199	0	0
0	0	0	237	0
459	0	0	0	0
0	0	0	0	0
0	0	0	0	0
160	109	0	0	228
0	0	0	0	0
0	0	0	121	224
0	0	739	0	0
0	0	0	0	0
0	0	0	0	0
0	0	0	0	0

ARDGGDY	ARDGGCDY	ARDGDY	ARDGDSSGYHFDY	ARDGDAFDI	ARDGAFDI
0	0	0	0	0	0
0	0	0	0	0	0
0	0	182	0	0	0
123	0	0	228	0	0
0	0	111	0	0	0
0	263	0	0	0	0
0	0	0	0	0	386
0	0	0	0	169	0
0	121	0	0	152	0
230	0	0	0	0	0
0	0	0	0	0	0
0	0	0	0	0	0
0	0	0	0	0	0
0	0	0	1156	0	246

ARDGAAAGLDY	ARDFSSGWYYFDY	ARDFGDY	ARDEGFDY	ARDCSGGSCYYY
0	0	0	0	0
0	0	0	0	0
156	0	0	0	0
0	0	0	0	0
0	0	0	0	0
0	0	228	0	3487
131	0	0	339	0
0	0	109	0	0
0	555	0	426	0
0	0	0	0	0
0	0	0	0	378
0	0	0	0	0
0	0	0	0	0
0	218	0	0	0

ARDAFDI	ARAYSSSLDY	ARAYGSGSYYYYGMDV	ARAVDTAMDY
0	0	0	0
0	0	0	0
0	0	0	0
0	0	0	175
0	0	0	0
0	0	0	185
0	0	0	0
0	177	126	0
273	0	0	0
0	133	145	0
0	0	0	0
0	0	0	0
0	0	0	0
663	0	0	0

ARAVAGTWWFDP	ARASSSWSYFDY	ARARYSSSWYFDY	ARAPPYYYYGMDV
0	0	0	0
0	0	0	0
0	0	0	0
0	0	0	0
0	0	0	0
0	228	206	0
113	0	0	433
371	0	0	0
0	0	0	167
0	0	0	0
0	0	197	0
0	0	0	0
0	0	0	0
0	1024	0	0

ARAHY	ARAGYSSGWYFDY	ARAGSYYYYGMDV	ARAGDGYNSNFDY	ARAGDAFDI
0	0	0	0	0
0	0	0	0	0
0	0	0	3336	0
0	0	114	0	0
0	103	0	0	133
0	0	256	0	0
0	0	0	0	254
0	0	0	0	0
312	159	0	0	0
0	0	0	267	0
335	0	0	0	0
0	0	0	0	0
0	0	0	0	0
0	0	0	0	0

ARAFDP	ARAFDI	ARADY	ARADWFDP	ARADSSSWYYFDY	ARADFDY
0	0	0	0	0	0
0	0	0	0	0	0
0	0	0	0	0	0
0	0	0	0	289	0
0	0	0	0	0	0
0	0	0	0	0	0
0	0	188	0	122	0
0	194	0	143	0	118
0	0	0	0	0	0
540	0	0	0	0	0
0	0	232	0	0	128
0	0	0	0	0	0
0	0	0	807	0	0
275	113	0	0	0	0

ARAAAGTSYYFDY	AKSPMDV	AKSGVVDY	AKSDWFDP	AKNYYDSSGYYYFDY
0	0	0	0	0
0	0	0	0	0
121	0	0	0	0
0	0	0	0	0
0	0	0	1777	0
0	0	0	0	0
0	0	226	0	0
0	0	0	0	0
0	0	0	9917	365
0	0	0	0	0
0	326	206	0	0
0	0	0	0	0
807	0	0	0	0
0	350	0	0	777

AKGYYDILTGYFDY	AKGPDAFDI	AKGLDY	AKGGVYFDY	AKGFDP	AKEYYYYGMDV
0	0	0	0	0	0
0	0	0	0	0	0
0	156	0	0	0	0
0	0	0	0	123	0
0	0	0	0	0	0
128	0	0	0	0	0
0	263	179	414	0	829
0	0	0	101	0	0
228	0	0	0	114	0
0	0	0	0	0	0
0	0	894	0	0	0
0	0	0	0	0	0
0	0	0	0	0	0
0	0	0	0	0	218

AKDYYYDSSGYFDY	AKDYYDSSGYFDY	AKDYGSGSYYFDY	AKDSYYDSSGYFDY
0	0	0	0
0	0	0	0
0	0	0	0
0	0	0	0
0	0	0	0
0	0	0	0
0	0	0	0
0	0	0	270
182	152	0	0
0	0	1124	0
0	0	0	0
0	0	0	0
0	0	0	680
568	1507	199	0

AKDSSGWYFDY	AKDSGSYSFDY	AKDSGD	AKDSDAFDI	AKDRVSSSWYYFDY
0	0	0	0	0
0	0	0	0	0
0	0	0	0	0
0	0	0	0	0
0	1110	162	0	0
0	0	0	0	0
0	0	0	0	0
0	0	0	0	0
700	0	0	129	0
0	0	0	0	103
0	447	163	0	275
0	0	0	0	0
0	0	0	0	0
132	0	0	1877	0

AKDRSYYYYGMDV	AKDRNYYYYGMDV	AKDRGYSYGPFDY	AKDRGYSSSWYYFDY
0	0	0	0
0	0	0	0
0	0	0	0
0	175	0	0
0	0	0	0
0	0	164	0
263	179	0	0
0	0	253	0
175	0	0	121
0	0	0	0
0	0	0	292
0	0	0	0
0	0	0	0
0	0	0	0

AKDRGLYYFDY	AKDRGGSYFDY	AKDQSSSWYYFDY	AKDPGGY	AKDPGDV
0	0	0	0	0
0	0	0	0	0
104	0	0	0	0
0	123	0	0	114
0	0	118	0	0
0	0	0	0	0
160	791	320	0	0
0	0	0	0	0
0	0	0	0	0
0	0	0	243	0
0	0	0	0	189
0	0	0	0	0
0	0	0	2342	0
0	0	0	0	0

AKDPFNYDYSNWFDP	AKDLVY	AKDLTQYNV	AKDLQFDY	AKDLNYYYYGMDV
0	0	0	0	0
240	0	0	0	0
0	0	0	0	0
0	0	0	0	0
0	0	5583	0	0
0	0	0	0	0
0	0	0	160	0
0	0	0	0	0
0	0	0	0	319
273	0	267	0	0
0	223	0	0	0
0	0	0	0	0
0	0	0	646	0
0	293	0	0	862

AKDKGGYLFDY	AKDIRNYYYGMDV	AKDIGGFDY	AKDHDSSGYYLYYFDY
0	0	0	0
0	0	0	0
0	0	0	0
0	0	0	0
0	0	0	0
0	164	0	0
0	0	0	0
0	0	143	0
228	0	213	144
0	133	0	133
0	0	0	0
0	0	0	0
0	0	0	0
113	0	0	0

AKDGYSSGWYYFDY	AKDGY	AKDDYYYYGMDV	AKASRDGYNYYFDY	AKANFDY
0	0	0	0	0
0	0	0	0	0
0	112	0	0	0
0	0	0	0	0
0	0	0	0	0
0	0	0	0	0
0	0	668	0	489
0	0	0	0	0
190	0	0	296	342
127	182	0	200	0
0	0	257	0	0
0	0	0	0	0
0	0	0	0	0
0	0	0	0	0

AKAHSSGWYYFDY	AKAFDY	AILLGY	AILDY	AHIVGGGNSGYFQH	AGGIAAALDY
0	0	0	0	0	0
0	0	0	0	0	0
0	0	0	182	0	0
0	0	0	0	0	0
0	0	0	0	0	0
0	0	0	0	0	242
263	0	169	0	0	0
0	0	0	135	160	0
0	106	0	0	0	0
0	0	279	0	0	0
0	240	0	0	0	292
0	0	0	0	0	0
219	0	0	0	0	0
0	0	0	0	180	0
